# Draft Genome Sequence of Clostridium botulinum Strain 277-00 Type B2

**DOI:** 10.1128/genomeA.00211-15

**Published:** 2015-04-02

**Authors:** Christelle Mazuet, Christiane Bouchier, Michel-Robert Popoff

**Affiliations:** a Institut Pasteur, Bactéries Anaérobies et Toxines, Paris, France; b Institut Pasteur, Plateforme Génomique, Paris, France

## Abstract

We report the draft genome sequence of Clostridium botulinum strain 277-00, which encodes a botulinum neurotoxin B2 associated with a *ha* gene locus. Strain 277-00 was isolated from a cheese responsible for an outbreak of botulism in Iran in 1997. This strain is closed to the bivalent B2/FA strain IBCA10-7060.

## GENOME ANNOUNCEMENT

Botulism is a rare but severe disease mainly resulting from food poisoning or intestinal colonization, which is characterized by flaccid paralysis leading to severe respiratory distress and death. Botulism is due to potent neurotoxins called botulinum neurotoxins (BoNTs), which are produced by Clostridium botulinum and atypical strains of Clostridium baratii and Clostridium butyricum (1).

Here, we report the draft genome sequence of strain 277-00 obtained by using a whole-genome sequencing (WGS) strategy with an insert length of about 526 bp on average, observed on the Fragment Analyzer Auto CE system (AATI). The WGS library was performed using the NextFlex PCR-free DNA sequencing kit (Bioo Scientific). The library was then sequenced on a HiSeq2500 machine in paired-end reads of 108 bases (Illumina). Sequence files were generated using Illumina Analysis Pipeline version 1.8 (CASAVA). After quality filtering, 2 million reads were assembled using CLC software version 4 (CLC Bio), yielding 91 scaffolds >1,000 bp with an average coverage about 110× and an *N*_50_ of 133,135 bases.

C. botulinum strain 277-00 was isolated from an outbreak of botulism due to ingestion of cheese in Iran (district of Qazvin) in 1997 (2, 3). WGS shows that strain 277-00 is a group I strain possessing a *bont/B2* gene within a hemagglutinin (*ha*) gene locus close to that of the C. botulinum B2 strain Prevot594 (4). However, the two strains belong to a unique and distinct MLST profile type: ST55 for strain 277-00 and a not-yet-reported MLST type in the Clostridium botulinum MLST database for strain Prevot594, which is close to ST54 associated with the French isolates C. botulinum B2 (F11, 2345) and C. sporogenes (283-98, 284-98).

Interestingly, strain 277-00 has also been found to be related to the recently characterized bivalent strain IBCA10-7060, which contains a *ha*-*bont/B2* locus and a *bont/FA* gene within an OrfX locus (5). In contrast to IBCA10-7060, strain 277-00 contains only the *ha-bont/B2* locus. Preliminary comparison of the sequence contigs of both strains shows that strain 277-00 contains a ~30-kbp segment, with numerous open reading frames (ORFs) predicted to be phage proteins, which are missing in IBCA10-7060. Conversely, contig 5 of IBCA10-7060, which harbors the *bont/FA* gene locus, is lacking in strain 277-00. BLAST analysis reveals that contig 5 of IBCA10-7060 exhibits 76% nucleotide sequence identity with plasmid pCLJ of strain Ba4 657 and 99% with plasmid pCBF from the bivalent A2B5 strain CDC1436. This result strongly suggests that the *bont/FA* gene locus is localized on plasmid in strain IBCA10-7060, which is missing in strain 277-00. Moreover, strain 277-00 contains additional ORFs scattered through the different contigs, which correspond to phage proteins according to the automatic genome annotation server (RAST) and which are lacking in IBCA10-7060.

Thereby, strain 277-00, albeit closely related to IBCA10-7060, shows a unique *ha-bont/*B2 locus and multiple DNA rearrangements such as probable acquisition/loss of phage/plasmid DNAs.

### Nucleotide sequence accession numbers.

This whole-genome shotgun project has been deposited at DDBJ/EMBL/GenBank under the accession number JXMY00000000. The version described in this paper is version JXMY01000000.
